# Baseline thrombopoietin level is associated with platelet count improvement in thrombocytopenic chronic hepatitis C patients after successful direct-acting antiviral agent therapy

**DOI:** 10.1186/s12876-021-01606-x

**Published:** 2021-01-21

**Authors:** Yen-Chun Chen, Ping-Hung Ko, Chi-Che Lee, Chih-Wei Tseng, Kuo-Chih Tseng

**Affiliations:** 1 Department of Internal Medicine, Dalin Tzu Chi Hospital, Buddhist Tzu Chi Medical Foundation, Chia-Yi, Taiwan No 2, Ming-Shen Road, Dalin Town, Chia-Yi County, 622 Taiwan; 2 School of Medicine, Tzu Chi University, Hualien, Taiwan; 3 Department of Medicine Research, Dalin Tzu Chi Hospital, Buddhist Tzu Chi Medical Foundation, Chia-Yi County, Taiwan

**Keywords:** Chronic hepatitis C, Direct-acting antiviral agents, Platelet, Thrombocytopenia, Thrombopoietin

## Abstract

**Background:**

Thrombocytopenia can rapidly improve in chronic hepatitis C (CHC) patients receiving direct-acting antiviral agents (DAA). The role of baseline (BL) thrombopoietin (TPO) in this phenomenon is unclear.

**Methods:**

From June 2016 to February 2019, a total of 104 CHC patients receiving DAA, with a sustained virologic response and BL thrombocytopenia, at Dalin Tzu Chi Hospital, were enrolled in this retrospective study. Significant platelet count improvement and platelet count improvement ratio were analyzed for correlation with BL TPO.

**Results:**

This cohort included 40 men (38.5%). Seventy-two (69.2%) patients had advanced fibrosis. The platelet count [median (range)] increased from 110.5 (32–149) × 10^3^/µL at BL to 116.5 (40–196) and 118.0 (35–275) × 10^3^/µL at end of treatment (EOT) and 12 weeks after EOT (P12), respectively, (EOT vs. BL, *P* < 0.001; P12 vs. BL, *P* < 0.001). BL TPO was positively correlated with significant platelet count improvement (*P* < 0.001), platelet count improvement ratio at EOT (*P* = 0.004), and P12 (*P* < 0.001). The area under the receiver operating characteristic curve and optimal cutoffs (pg/ml) were 0.77 (95% confidence interval, 0.67–0.86) and 120, respectively, for significant platelet count improvement prediction. The sensitivity, specificity, and accuracy were 88.6%, 71.7%, and 78.8%, respectively.

**Conclusions:**

BL TPO level might be a useful marker for predicting significant platelet count improvement in thrombocytopenic patients after successful DAA therapy.

## Background

Thrombocytopenia (defined as < 150 × 10^3^/μL) in patients with chronic liver disease (CLD) is relatively common, especially in chronic hepatitis C (CHC)-infected patients. The prevalence is about 6% in CLD patients, 24% in CHC patients, and up to 78% in cirrhotic patients [1–3]. Sustained virologic response (SVR), achieved by interferon-based therapy, for CHC-related advanced liver fibrosis improves thrombocytopenia after a long-term follow-up [4, 5]. This was thought to be associated with an improved fibrosis stage and portal hypertension [4, 5]. Reportedly, thrombocytopenia rapidly improves in CHC patients receiving direct-acting antiviral agents (DAA) [6–10]. However, this improvement, within short-term follow-up, is less likely due to changes in the fibrosis stage [11]. It suggests that factors other than fibrosis may play important roles, such as hypersplenism, thrombopoietic cytokines, antiplatelet antibody, or direct effect from hepatitis C virus (HCV) via suppressing hematopoiesis [3, 12–17].

Thrombopoietin (TPO), a thrombopoietic cytokine, is involved in megakaryocyte maturation and platelet production [18]. TPO is primarily produced in the liver and its level is affected by liver fibrosis stage and platelet turnover [3, 13–15, 19]. This partly accounts for thrombocytopenia in CLD patients [3]. TPO mimetics have been shown to improve platelet count in thrombocytopenic CLD patients [20].

Data on the relationship between TPO and rapid thrombocytopenia improvement after DAA therapy has not been well reported. Hence, this study investigated the association between baseline (BL) TPO and platelet count improvement after HCV clearance.

## Methods

### Patient selection

**Fig. 1:**
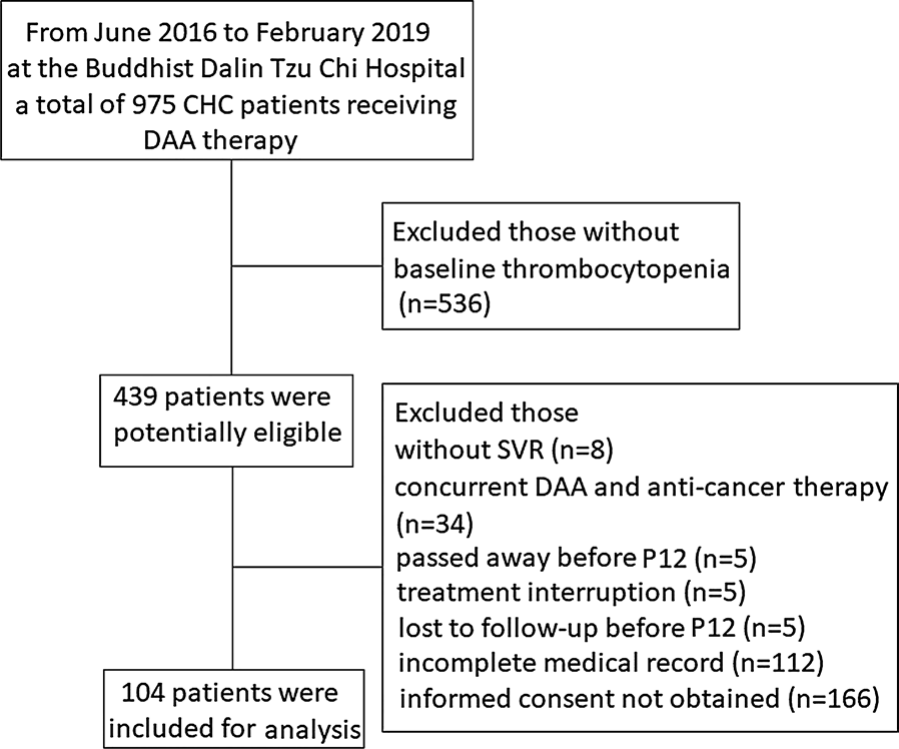
Flow diagram used in building this study cohort

CHC-infected patients with BL thrombocytopenia (platelet count < 150 × 10^3^/µL), who underwent DAA treatment with SVR at Dalin Tzu Chi Hospital from June 2016 to February 2019, were screened in this retrospective study. All patients had been positive for anti-hepatitis C antibody for more than 6 months and had detectable serum HCV RNA levels at the time of entry into the study. Treatment duration and regimen were based on guidelines [21–24]. Patients without SVR, patients with concurrent DAA and anticancer therapy, patients who passed away before 12 weeks after end of treatment (P12), patients with treatment interruption, patients lost to follow-up before P12, patients with incomplete medical records, or patients without informed consent were excluded. A total of 104 patients were enrolled in the study (Fig. 1). The study conformed to the ethical guidelines of the Declaration of Helsinki 1975, with prior approval by the Ethics Committee of Dalin Tzu Chi Hospital (approval number B10704013). Informed consent was obtained.

### Clinical monitoring

For all patients, blood tests, including serum aspartate aminotransferase (AST), alanine aminotransferase (ALT), total bilirubin, creatinine, hemoglobin, white blood cell, and platelet counts, and HCV RNA, were checked at the hepato-gastrointestinal outpatient clinic at BL, end of treatment (EOT), and P12. We defined significant platelet count improvement as a > 10% platelet count improvement ratio at P12, compared to that at BL. The platelet count improvement ratio was defined as [platelet count (EOT or P12)–platelet count (BL)]/platelet count (BL).

Abdominal sonography was also conducted before, during, or after DAA treatment as recommended [21, 24]. Hepatic fibrosis was evaluated using the noninvasive fibrosis-4 (FIB-4) test [22]. Advanced fibrosis was defined as FIB-4 > 3.25 [22]. Fatty liver diagnosis was based on abdominal ultrasound results, including hepatorenal echogenicity contrast, liver brightness, deep attenuation, and vessel blurring [25]. Splenomegaly was diagnosed when the spleen length of the long axis was more than 11 cm [26]. All ultrasonographic images were stored as photographs.

Other clinical factors, including chronic hepatitis B status, hepatocellular carcinoma (HCC), and alcoholism, were recorded by chart review. Alcoholism was defined as alcohol consumption of more than 40 g/day [27]. HCC was diagnosed either by biopsy or by imaging in the setting of liver cirrhosis [23]. Chronic hepatitis B was diagnosed if a patient had seropositivity for the hepatitis B surface antigen for at least 6 months.

### HCV quantification and genotyping

Serum HCV RNA was quantified at BL, EOT, and P12 using the COBAS AmpliPrep/COBAS TaqMan HCV Test, v2.0 (Roche Diagnostics, Rotkreuz, Switzerland), with a lower limit of quantification of 15 IU/mL. HCV genotyping was performed using the COBAS HCV GT (Roche Diagnostics).

### Thrombopoietin assay

TPO was measured in BL serum samples with a sandwich enzyme-linked immunosorbent assay (ELISA; FineTest, Wuhan, China), following the manufacturer’s recommendations [28]. Samples were stored at − 40 °C until analyzed. The lower detection limit of the TPO assay was 18.75 pg/mL. Additionally, quality control samples were included in each assay.

### Statistical analyses

The commercial statistical software package (SPSS for Windows, version 22) was used for all statistical analyses. Continuous variables were presented as median and range. The chi-square or the Fisher’s exact test was used for nominal variables. Continuous variables were compared using the Student’s *t*-test or the Mann–Whitney U test, when applicable for two independent groups. The Spearman’s rank correlation coefficient was used to examine factors associated with platelet count improvement. A receiver operating characteristic (ROC) curve was used to calculate the TPO predictive value for patients with significant platelet improvement. *P* < 0.05 was considered significant in all analyses.

## Results

### Baseline characteristics of thrombocytopenic CHC patients

A total of 104 patients were included in the analysis. BL characteristics are shown in Table 1. This cohort included 40 men (38.5%) and the median age was 66.1 (60.1–74.2) years. Most patients were infected with the HCV genotype 1 (n = 71, 68.3%). Of the patients in the cohort, 72 had advanced fibrosis (69.2%) and 28 had a history of HCC (26.9%). Sofosbuvir/ledipasvir (SOF/LDF) (n = 34, 32.7%), paritaprevir/ritonavir/ombitasvir with dasabuvir (n = 27, 26.0%), elbasvir/grazoprevir (EBR/GZR) (n = 10, 9.6%), and sofosbuvir/daclatasvir (n = 9, 8.7%) were the most commonly used DAAs.

**Table 1:** Baseline characteristics of chronic hepatitis C patients with thrombocytopenia with or without significant platelet count improvement

Variable	All patients (n = 104)	With significant platelet count improvement (n = 44) (42.3%)	Without significant platelet count improvement (n = 60) (57.7%)	*P*-value
Age (years)^a^	66.1 (37–88)	64.4 (46–84)	67.8 (37–88)	0.33
Male (%)	40 (38.5%)	12 (27.3%)	28 (46.7%)	0.066
Alcoholism (%)	5 (4.8%)	2 (4.5%)	3 (5%)	1.00
Fatty liver	29 (27.9%)	14 (31.8%)	15 (25.5%)	0.510
ALT(U/L)^a^	75 (20–492)	71.5 (20–448)	75.0 (20–492)	0.603
AST(U/L)^a^	54 (10–431)	43.5 (10–410)	58.5 (23–431)	0.194
Albumin (g/dL)^a^	4.1 (3–5)	4.0 (3–5)	4.1 (3–5)	0.532
Total bilirubin (mg/dL)^a^	0.80 (0–3)	0.9 (0–2)	0.8 (0–3)	0.971
e-GFR (ml/min/1.73 m^2^)^a^	75.7 (4.5–132.1)	75.5 (4.5–118.0)	76.0 (11.9–132.1)	0.594
Platelet (× 10^3^/µL)^a^	110.5 (32–149)	115.5 (32–149)	106.5 (41–148)	0.932
Prothrombin time (INR)^a^	1.1 (0.9–1.3)	1.1 (1.0–1.3)	1.1 (0.9–1.3)	0.203
AFP (ng/mL)^a^	6.0 (2–253)	7.2 (2–253)	5.1 (2–219)	0.064
Baseline HCV viral load (IU/mL)^a^	1.35 × 10^6^ (176–35.8 × 10^6^)	1.63 × 10^6^ (1890–32.8 × 10^6^)	1.13 × 10^6^ (176–35.8 × 10^6^)	0.453
HCV genotype 1 (%)	71 (68.3%)	32 (72.7%)	39 (65.0%)	0.523
Interferon experienced (%)	31 (29.8%)	16 (36.4%)	15 (25.0%)	0.278
HBV co-infection (%)	5 (4.8%)	2 (4.5%)	3 (5%)	1.00
FIB-4^a^	4.2 (1.05–20.17)	3.6 (1.05–19.92)	4.9 (1.32–20.17)	0.175
Advanced fibrosis (%)	72 (69.2%)	26 (59.1%)	46 (76.7%)	0.085
HCC history (%)	28 (26.9%)	10 (22.7%)	18 (30.0%)	0.504
Splenomegaly (%)	38 (36.5%)	12 (27.3%)	26 (43.3%)	0.104
Ascites (%)	9 (8.4%)	1 (2.3%)	8 (12.7%)	0.075
TPO (pg/mL)^a^	132.3 (32.0–3944.0)	172.2 (70.0–3944.0)	74.8 (32.0–3850.0)	< 0.001

ALT, alanine aminotransferase; AST, aspartate aminotransferase; eGFR, estimated glomerular filtration rate; AFP, alpha-fetoprotein; FIB-4, fibrosis-4; HCC, hepatocellular carcinoma; TPO, thrombopoietin

^a^Data are expressed as median (range)

### Clinical factors associated with significant platelet count improvement

**Fig. 2:**
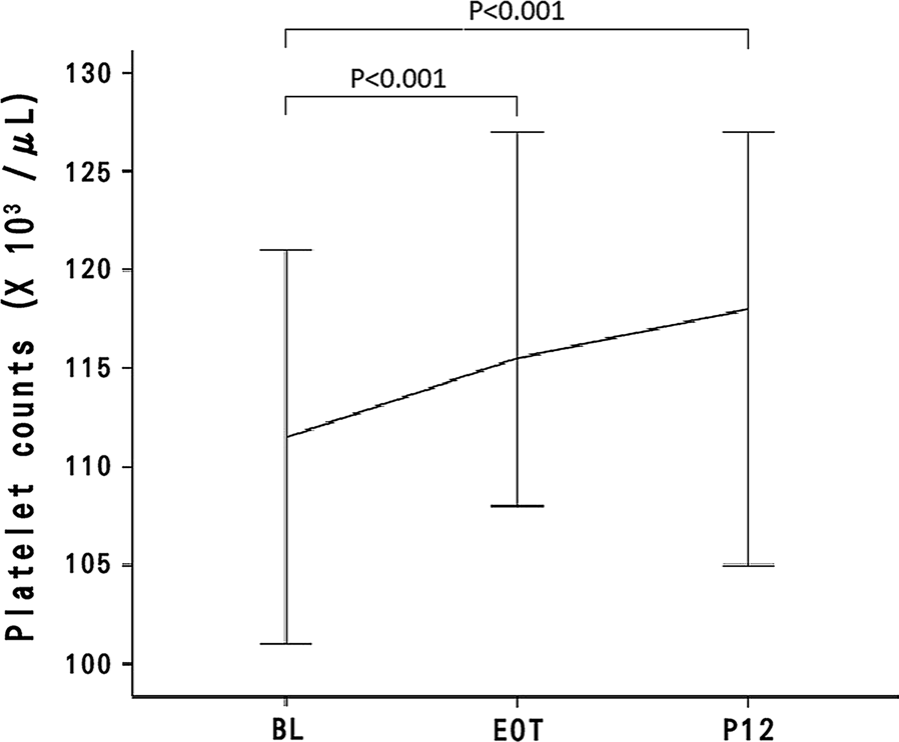
Overall platelet count change (median with confidence interval) at BL, EOT, and P12. The median (range) platelet count was 110.5 (91.2–132.7) × 10^3^/µL at BL and increased to 116.5 (90.7–147.2) × 10^3^/µL and 118.0 (90.5–149.0) × 10^3^/µL at EOT and P12, respectively. It was statistically significant at EOT and P12, compared to the BL platelet levels (EOT vs. BL, *P* < 0.001 and P12 vs. BL, *P* < 0.001). BL: baseline; EOT: end of treatment; and P12: 12 weeks after EOT

Changes in the median platelet count from BL to P12 are shown in Fig. 2. The median(range) platelet count was 110.5 (91.2–132.7) × 10^3^/µLat BL and increased to 116.5 (90.7–147.2) and 118.0 (90.5–149.0) × 10^3^/µL at EOT and P12, respectively. The platelet level was statistically significant at EOT and P12, compared to that at BL (EOT vs. BL, *P* < 0.001; P12 vs. BL, *P* < 0.001).

Forty-four patients (42.3%) showed significant platelet count improvement. The BL characteristics of patients with and without significant platelet count improvement are shown in Table 1. No variables, including age, gender, alcoholism, presence of fatty liver, liver function tests, HCV viral load, advanced hepatic fibrosis status, and previous HCC history, were significantly different between both groups. BL platelet count [115.5 (32–149) vs.106.5 (41–148) × 10^3^/µL, *P* = 0.932) was also not significantly different between both groups.

### Factors associated with baseline TPO levels

BL TPO levels in CHC patients, with and without significant platelet count improvement, were 172.2 (70–3944) and 74.8 (32–3850) pg/ml, respectively (Table 1). The difference was statistically significant (*P* < 0.001). The TPO level in those with and without splenomegaly was 79.1 (32–1247) and 148.75 (40–3944) pg/ml (*P* = 0.002), respectively. The TPO level between advanced and non-advanced hepatic fibrosis was 115.7 (38–3944) vs. 133.1 (32–3644) pg/ml (*P* = 0.719), respectively.

**Fig. 3:**
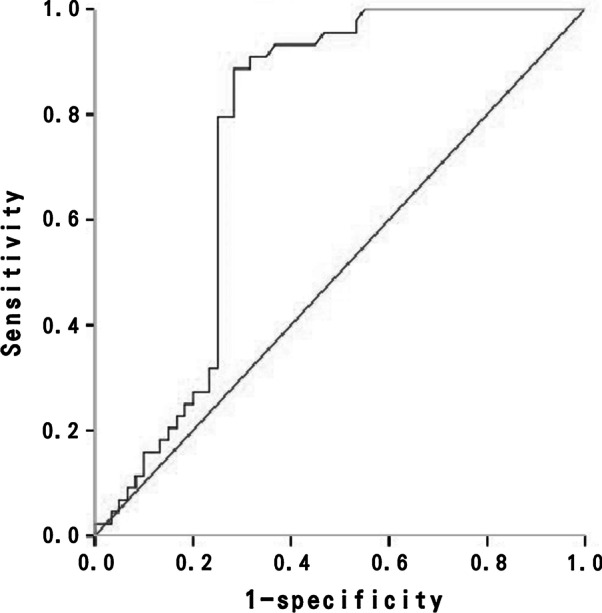
Receiver operating characteristic (ROC) curve of thrombopoietin (TPO) stratifying CHC-related thrombocytopenic patients, with significant platelet count improvement. Area under the curve for TPO in these patients was 0.765 (95% confidence interval, 0.670–0.861)

The BL TPO was positively correlated with the platelet count improvement ratio at EOT (ρ = 0.282, *P* = 0.004) and P12 (ρ = 0.502, *P* < 0.001). The BL TPO levels was also positively correlated with significant platelet count improvement in different genotype (genotype 1: *P* < 0.001; non-genotype 1: *P* = 0.016) and liver fibrosis severity (FIB-4 ≤ 3.25: *P* = 0.002; FIB-4 > 3.25: *P* = 0.001). We used ROC analysis to test TPO levels to determine the cutoff value for predicting significant platelet count improvement among these patients (Fig. 3). The area under the ROC curve for TPO to predict platelet count improvement was 0.765 (95% confidence interval, 0.670–0.861). TPO levels higher than 120 pg/ml could predict significant platelet count improvement, with sensitivity, specificity, and accuracy of 88.6%, 71.7%, and 78.8%, respectively.

## Discussion

This study showed that overall platelet counts were significantly increased in thrombocytopenic CHC patients after successful DAA treatment. BL TPO is positively associated with significant platelet count improvement and platelet count improvement ratio. BL TPO level > 120 pg/ml can predict significant platelet count improvement.

BL TPO can be positively associated with significant platelet count improvement. BL TPO is also correlated with platelet count improvement ratio at EOT and P12 (ρ = 0.282, *P* = 0.004 and ρ = 0.502, *P* < 0.001 at EOT and P12, respectively). In addition, this positive association existed both in different genotypes (genotype 1 and non-genotype 1 in this study) and different liver fibrosis severity (non-advanced fibrosis: FIB-4 ≤ 3.25 and advanced fibrosis: FIB-4 > 3.25). This association between BL TPO and platelet count improvement may be related to the splenic size and fibrosis stage. Previous studies have demonstrated that a higher TPO is related to a smaller spleen size [29, 30] and less hepatic fibrosis [13–15, 19, 31]. In our study population, it was shown that those without splenomegaly had significantly higher TPO levels. However, analysis of the TPO levels between non-advanced and advanced fibrosis patient groups showed no significant difference. TPO alone may not truly reflect synthesis function of the liver because it is produced by the liver at a constant rate and removed from circulation by TPO receptors on megakaryocytes and platelets [32, 33]. Platelet turnover also affects TPO level [3, 34]. We did not check platelet turnover; therefore, the low TPO level might be attributed to either poor liver function or high platelet turnover. Another likely reason is that we classified non-advanced or advanced hepatic fibrosis by the FIB4 score; some patients in the advanced fibrosis group may actually have non-advanced hepatic fibrosis. The actual reason requires further studies checking platelet turnover and using liver biopsy as a classification method for TPO level analysis in different fibrosis stages.

Though we knew baseline TPO was positively correlated to the platelet count change, the cut-off value was unclear. Therefore, we used ROC test and found the baseline TPO level > 120 pg/ml was associated with significant platelet count improvement. The sensitivity is 88.6%, and specificity is 71.7%, with accuracy being 78.8%. Hence, when thrombocytopenia is observed in untreated CHC patients, this cut-off value may be helpful in predicting who would have significant improvement after DAA therapy within short-term follow-up (P12).

In the current study, BL TPO levels were not correlated with BL platelet count (ρ = 0.054, *P* = 0.587). This result was also corroborated by other studies [19, 29, 30]. It is reasonable to see this phenomenon because absolute platelet count is affected by TPO, spleen size, autoimmune status, megakaryocyte mass in bone marrow, and the aforementioned viral effect [16, 29]. We also found that the TPO level (32–3944 pg/mL) in patients was more variable, compared to the normal population (81.3–237.7 or 19.25–377.75 pg/ml) [29, 35]. This suggests that the ability of our patients to produce or destroy TPO differed significantly. Although some patients, with liver disease, had higher TPO levels, the elevation was mostly inadequate, compared with thrombocytopenic patients due to other diseases such as aplastic anemia or chemotherapy-induced thrombocytopenia [31]. In patients with early stage liver fibrosis, TPO production may increase to partly compensate for the decreased platelet count; those with more advanced fibrosis or liver cirrhosis produce relatively and inappropriately low TPO [13–15, 19, 31]. Moreover, other factors such as hypersplenism, may offset the positive effect of elevated TPO. Congested spleen contains a large platelet mass, which would bind to, internalize, and destroy TPO [30, 36]. We found that those with splenomegaly had significantly lower TPO than those without splenomegaly. A negative association between TPO level and spleen size was also reported by other studies [29, 30], but there was a study revealing no correlation between them [19]. These differences may be due to the relatively small number of cases in these studies and patient selection criteria.

This study found that the only factor associated with significant platelet count improvement was high BL TPO. Other BL characteristics were not related to such changes after DAA therapy. This was partly different from our previous study [8]. Our previous study showed that moderate to severe fatty liver and low BL platelet count could predict significant platelet count improvement. The most likely reasons were that some differences existed in the previous study, compared to findings of the present study, including more case numbers (n = 249), more cancer patients (n = 19 vs. n = 0, *P* = 0.001), viable HCC patients (n = 14 vs. n = 0, *P* = 0.013), more advanced hepatic fibrosis patients (n = 213 vs. n = 72, *P* = 0.001), and more moderate to severe fatty liver patients (n = 24 vs. n = 5, *P* = 0.2). Although there were not statistically significant, patients with advanced hepatic fibrosis status, higher FIB-4 scores, HCC history, splenomegaly, and ascites would have a trend without significant platelet count improvement. These statistically insignificant difference may be due to a relatively small patient sample size in this study.

There are some limitations of our study. First, we did not perform biopsy of the liver in all patients to check inflammation and fibrosis severity. Instead, we used noninvasive indices such as the FIB-4 test, to identify patients with advanced fibrosis; this is a relatively reliable method [22]. But false positive results may exist. Therefore, other invasive method like biopsy or noninvasive method such as vibration-controlled transient liver elastography should be considered as part of fibrosis assessment in the future studies. Second, we did not perform bone marrow biopsy in this study to exclude possible indolent hematological disorders in our patients. Bone marrow biopsy is an invasive procedure, which could not be performed except with a clinically strong indication. Third, we did not check for platelet turnover. However, hypersplenism is the main contributor to platelet turnover in patients with splenomegaly [34], which were included in our study. In patients without splenomegaly, more evidence is required to clarify the association between platelet count and turnover, TPO level, and liver fibrosis severity. Fourth, other thrombopoietic cytokines such as interleukin-6, -11, and-3, were not evaluated. Their importance among these thrombocytopenic patients remains unknown. Therefore, further studies are needed. Fifth, checking the baseline TPO levels in patients with severe thrombocytopenia may have a stronger impact on clinical practice and might be more cost-effective. Hence, further study enrolling severe thrombocytopenic patients is required to confirm the role of TPO in such circumference. Sixth, the dynamic changes of TPO levels during and after DAA treatment were not investigated in this study. The correlation of BL TPO and dynamic TPO levels with long-term platelet count or long-term fibrosis status would be an interesting issue. Therefore, further study is needed to explore these associations.

## Conclusion

Successful DAA therapy for CHC patients results in significant platelet count improvement. BL TPO level is positively correlated with significant platelet count improvement and platelet count improvement ratio. The BL TPO cutoff value of 120 pg/ml can predict significant platelet count improvement among CHC-related thrombocytopenic patients.

## Data Availability

The datasets generated and analyzed during the current study are available from the corresponding author on reasonable request.

## Abbreviations

CLD: Chronic liver disease
CHC: Chronic hepatitis C
SVR: Sustained virologic response
DAA: Direct-acting antiviral agent
HCV: Hepatitis C virus
TPO: Thrombopoietin
BL: Baseline
P12: 12 Weeks after end of treatment
AST: Aspartate aminotransferase
ALT: Alanine aminotransferase
EOT: End of treatment
FIB-4: Fibrosis-4
HCC: Hepatocellular carcinoma
eGFR: Estimated glomerular filtration rate
AFP: Alpha-fetoprotein

## References

[1] Giannini EG, Afdhal NH, Sigal SH (2015). Non-cirrhotic thrombocytopenic patients with hepatitis C virus: characteristics and outcome of antiviral therapy. J Gastroenterol Hepatol.

[2] Louie KS, Micallef JM, Pimenta JM, Forssen UM (2011). Prevalence of thrombocytopenia among patients with chronic hepatitis C: a systematic review. J Viral Hepat.

[3] Peck-Radosavljevic M (2017). Thrombocytopenia in chronic liver disease. Liver Int.

[4] Kee KM, Wang JH, Hung CH, Chen CH, Lee CM, Lu SN (2013). Improvement of thrombocytopenia in hepatitis C-related advanced fibrosis patients after sustained virological response. Dig Dis Sci.

[5] van der Meer AJ, Maan R, Veldt BJ (2016). Improvement of platelets after SVR among patients with chronic HCV infection and advanced hepatic fibrosis. J Gastroenterol Hepatol.

[6] Rafei H, Ascensao JL, Aggarwal A (2016). Platelet count increase seen with Ledipasvir-Sofosbuvir combination treatment of chronic hepatitis C in patients with thrombocytopenia. Blood.

[7] Welzel TM, Petersen J, Herzer K (2016). Daclatasvir plus sofosbuvir, with or without ribavirin, achieved high sustained virological response rates in patients with HCV infection and advanced liver disease in a real-world cohort. Gut.

[8] Chen Y-C, Tseng C-W, Tseng K-C (2020). Rapid platelet count improvement in chronic hepatitis C patients with thrombocytopenia receiving direct-acting antiviral agents. Medicine.

[9] Ishizu Y, Ishigami M, Hayashi K (2020). Rapid increase of platelet counts during antiviral therapy in patients with hepatitis C virus infection. Hepatol Res.

[10] Soliman Z, El Kassas M, Elsharkawy A, et al. Improvement of platelet in thrombocytopenic HCV patients after treatment with direct-acting antiviral agents and its relation to outcome. *Platelets.* 2020:1–8. 10.1080/09537104.2020.1742313 32250721

[11] Ioannou GN, Feld JJ. What are the benefits of a sustained virologic response to direct-acting antiviral therapy for hepatitis C virus infection? *Gastroenterology.* 2019;156(2):446–460 e442. 10.1053/j.gastro.2018.10.033 30367836

[12] Kajihara M, Kato S, Okazaki Y (2003). A role of autoantibody-mediated platelet destruction in thrombocytopenia in patients with cirrhosis. Hepatology.

[13] Koike Y, Yoneyama A, Shirai J (1998). Evaluation of thrombopoiesis in thrombocytopenic disorders by simultaneous measurement of reticulated platelets of whole blood and serum thrombopoietin concentrations. Thromb Haemost.

[14] Giannini E, Borro P, Botta F (2002). Serum thrombopoietin levels are linked to liver function in untreated patients with hepatitis C virus-related chronic hepatitis. J Hepatol.

[15] Tana MM, Zhao X, Bradshaw A (2015). Factors associated with the platelet count in patients with chronic hepatitis C. Thromb Res.

[16] Moore AH (2019). Thrombocytopenia in cirrhosis: a review of pathophysiology and management options. Clin Liver Dis (Hoboken).

[17] Pascutti MF, Erkelens MN, Nolte MA (2016). Impact of viral infections on hematopoiesis: from beneficial to detrimental effects on bone marrow output. Front Immunol.

[18] Thrombopoietin KK (1998). N Engl J Med.

[19] Pradella P, Bonetto S, Turchetto S (2011). Platelet production and destruction in liver cirrhosis. J Hepatol.

[20] Olson SR, Koprowski S, Hum J, McCarty OJT, DeLoughery TG, Shatzel JJ (2019). Chronic liver disease, thrombocytopenia and procedural bleeding risk; are novel thrombopoietin mimetics the solution?. Platelets.

[21] European Association for Study of L. EASL Clinical practice guidelines: management of hepatitis C virus infection. *J Hepatol.* 2014;60(2):392–420. 10.1016/j.jhep.2013.11.003 24331294

[22] European Association for the Study of the Liver. Electronic address eee, European Association for the Study of the L. EASL Recommendations on Treatment of Hepatitis C 2018. *J Hepatol.* 2018;69(2):461–511. 10.1016/j.jhep.2018.03.026 29650333

[23] European Association for the Study of the Liver. Electronic address eee, European Association for the Study of the L. EASL Clinical Practice Guidelines: Management of hepatocellular carcinoma. *J Hepatol.* 2018;69(1):182–236. 10.1016/j.jhep.2018.03.019 29628281

[24] Panel AIHG (2015). Hepatitis C guidance: AASLD-IDSA recommendations for testing, managing, and treating adults infected with hepatitis C virus. Hepatology.

[25] Hamaguchi M, Kojima T, Itoh Y (2007). The severity of ultrasonographic findings in nonalcoholic fatty liver disease reflects the metabolic syndrome and visceral fat accumulation. Am J Gastroenterol.

[26] Frank K, Linhart P, Kortsik C, Wohlenberg H (1986). Sonographic determination of spleen size: normal dimensions in adults with a healthy spleen. Ultraschall Med.

[27] Stickel F, Datz C, Hampe J, Bataller R (2017). Pathophysiology and Management of Alcoholic Liver Disease: Update 2016. Gut Liver.

[28] Human TPO(Thrombopoietin) ELISA Kit https://static.fn-test.com/product/manuals/elisa/EH0422.pdf.

[29] Aref S, Mabed M, Selim T, Goda T, Khafagy N (2004). Thrombopoietin (TPO) levels in hepatic patients with thrombocytopenia. Hematology.

[30] Rios R, Sangro B, Herrero I, Quiroga J, Prieto J (2005). The role of thrombopoietin in the thrombocytopenia of patients with liver cirrhosis. Am J Gastroenterol.

[31] Nichol JL, Endogenous TPO (1998). (eTPO) levels in health and disease: possible clues for therapeutic intervention. Stem Cells.

[32] Kaushansky K (2006). Lineage-specific hematopoietic growth factors. N Engl J Med.

[33] Kuter DJ (2013). The biology of thrombopoietin and thrombopoietin receptor agonists. Int J Hematol.

[34] Salvagno GL, Montagnana M, Degan M (2006). Evaluation of platelet turnover by flow cytometry. Platelets.

[35] Singh A, Verma A, Nityanand S, Chaudhary R, Elhence P (2015). Circulating thrombopoietin levels in normal healthy blood donors and in aplastic anemia patients in relation to disease severity. Asian J Transfus Sci.

[36] Hitchcock IS, Kaushansky K (2014). Thrombopoietin from beginning to end. Br J Haematol.

